# Supplementary material for: Salt marshes create more extensive channel networks than mangroves
Source: Nat Commun. 2022 Apr 19;13:2017. doi: 10.1038/s41467-022-29654-1 (PMC9018726; doi:10.1038/s41467-022-29654-1)
Supplement: Supplementary file 1 — Supplementary Information [file 41467_2022_29654_MOESM1_ESM.pdf]

Nature Communications

Supplementary Information for

Salt marshes create more extensive channel networks than mangroves

Christian Schwarz[1,2,3,4], Floris van Rees[4,5], Danghan Xie[4], Maarten Kleinhans[4] and Barend van Maanen[6]*

[1] Department of Civil Engineering, KU Leuven, Kasteelpark Arenberg 40, B-3001 Leuven, Belgium
[2] Department of Earth and Environmental Sciences, KU Leuven, Leuven, 3000, Belgium,
[3] School of Marine Science and Policy, University of Delaware, Lewes, US
[4] Department of Physical Geography, Utrecht University, Utrecht, the Netherlands,
[5] Deltares, Rotterdamseweg 185, 2629 HD Delft, Netherlands
[6] College of Life and Environmental Sciences, University of Exeter, Exeter, UK

**E-mail: christian.schwarz@kuleuven.be*

Contents of this file

Supplementary Tables 1 to 3
Supplementary Figures 1 to 10

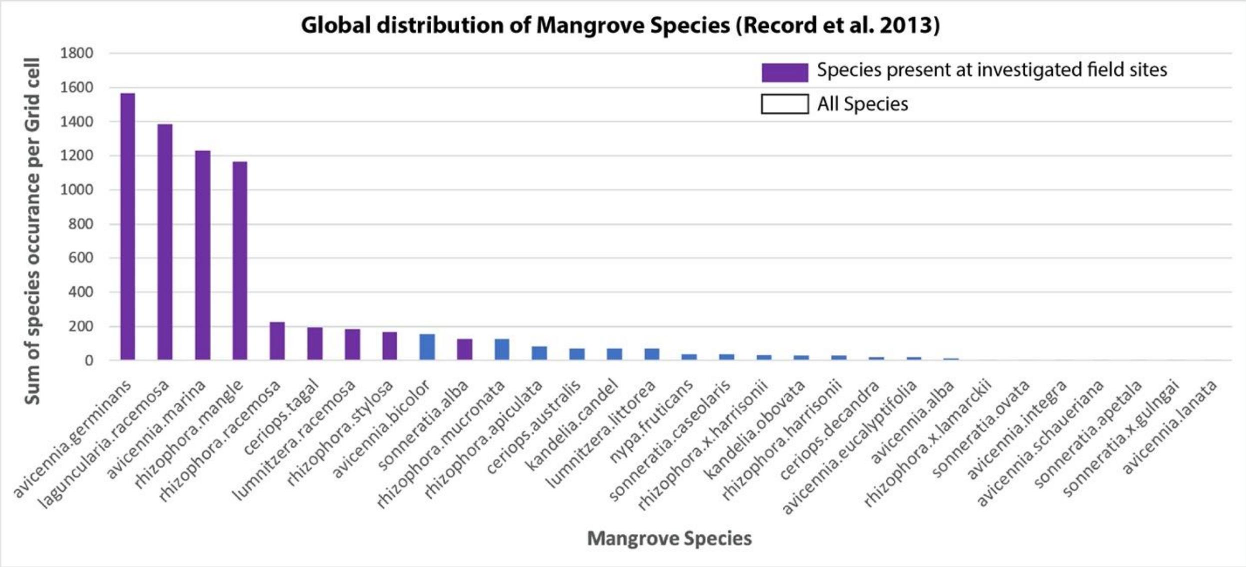

Supplementary Figure 1. Global Mangrove Species distribution:
Record et al. (2013) used species distribution modelling to assess global mangrove species distributions around low latitude coasts with a 2.5- minute resolution (4,318 m grid cells) in a Goode homolosine projection. All GBIF data within 40 km of the coastline were assigned to the nearest grid cell of the current coast; these occurrence records yielded 7,085 unique records distributed across 1,847 grid cells that were used in the models, which treated each coastal grid cell as an observation unit. Above we show the ranked sum of all species occurrences; purple color represents the species present at our field sites (Table S2). Vivipary exists in all species of the following four genera: *Rhizophora*, *Kandelia*, *Bruguiera* and *Ceriops*; Cryptovivipary occurs in *Avicennia* (*Avicenniaceae*) and *Nypa* (*Palmae*). Therefore, vivipary is well represented in different taxonomic groups in the mangrove ecosystems.

Record, S., Charney, N.D., Zakaria, R.M. and Ellison, A.M., 2013. Projecting global mangrove species and community distributions under climate change. Ecosphere, 4(3), pp.1-23.

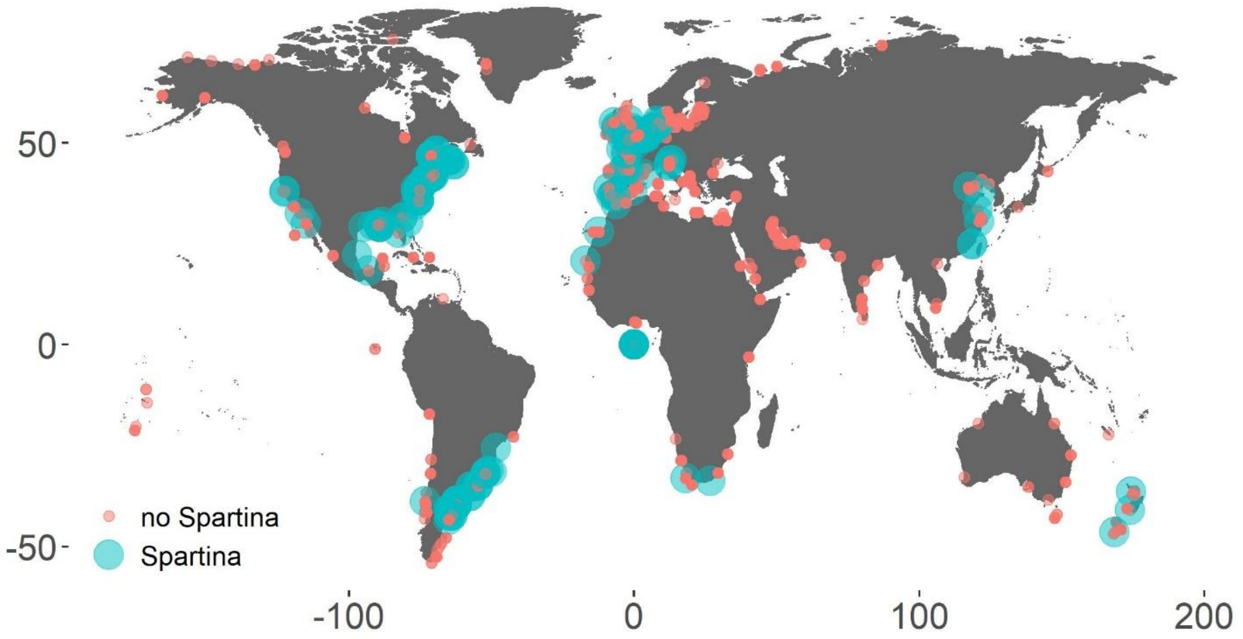

Supplementary Figure 2a. Global Distribution of the Genus Spartina (turquoise) relative to all salt marsh species surveyed (red) in McOwen et al. (2018).

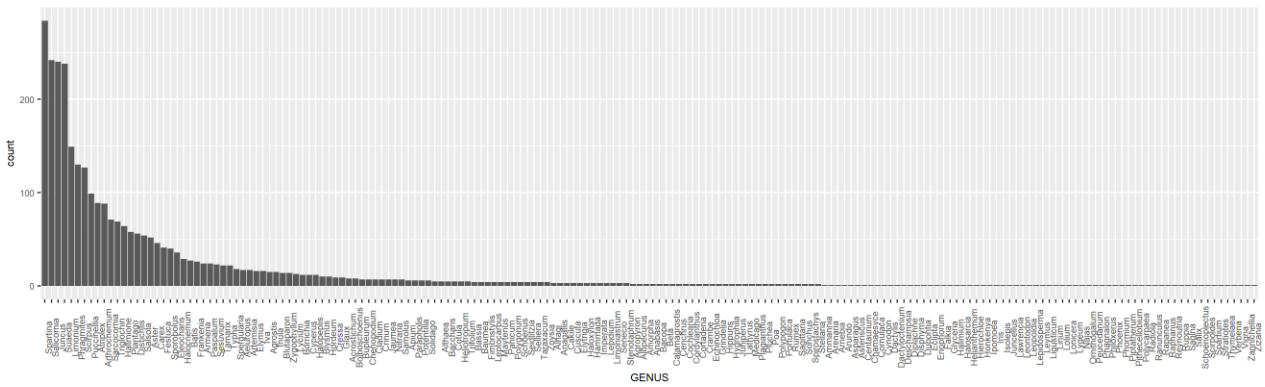

Supplementary Figure 2b. Frequency of global distribution of salt marsh species sorted by Genus in McOwen et al. (2018). The most frequent genus is *Spartina*.

McOwen, C. J., L. V. Weatherdon, J. W. Van Bochove, and others. 2017. A global map of saltmarshes. Biodivers. Data J. 5. doi:10.3897/BDJ.5.e11764.

Site	ID	Vegetation	Latitude, longitude (decimal degrees)	UTM zone	mtl (m)	mstl (m)	elev.(m rel. msl)	Source tidal range	Davies (1964) classification	Type	Date (dd/mm/yy)
Gulf of Nicoya, Costa Rica	M1	mangrove	10°7'N, 85°15'W	16N	2.3	2.8	1.2[e1]	Voorhis et al., 1983	Meso-tidal	Estuary*	8/6/2018
Mekong, Vietnam	M2	mangrove	9°31'N, 106°16'E	48N	2.2	3.2	0.8[e2]	Wolanski et al., 1996	Meso-tidal	Open/ Delta*	12/2/2018
Darwin Harbour, Australia	M3	mangrove	12°26' S, 130°52' E	52S	-	5.5	1.1[e3]	Woodroffe et al. , 1988	Macro-tidal	Estuary*	9/8/2018
Churute, Ecuador	M4	mangrove	2°37' S, 79°45' W	17S	8	-	1.8[e4]	Adame and Lovelock, 2011	Macro-tidal	Open/ Delta*	9/7/2018
Kala Oya, Sri Lanka	M5	mangrove	8°31' N, 79°84' E	44N	-	0.8	0.4[e5]	Jaccarini and Martens, 2013	Micro-tidal	Lagoon*	18/7/2018
Goodland, USA	M6	mangrove	25° 56' N, 81 58' W	17N	-	0.3	1.5[e6]	Simard et al., 2006	Micro-tidal	Lagoon*	14/7/2018
Whitianga, New Zealand	M7	mangrove	36°52'56'' S, 175°42''2' E	60S		2.2	0.75[e7]	Horstman et al. 2021	Meso-tidal	Lagoon[e7]	30/9/2021
Le Mont Saint Michel, France	S1	salt marsh	48°38' N, 1°34' W	30N	-	13.2	6.0[e8]	Tessier, 1993	Macro-tidal	Open/ Bay[e]	19/5/2018
Venice lagoon, Italy	S2	salt marsh	45°30' N, 12 22'E	33N	1	-	0.1[e9]	Tambroni and Seminara, 2006	Micro-tidal	Lagoon[d]	17/7/2018
Norfolk, United Kingdom	S3	salt marsh	52°51' N, 0°12'E	31N	-	3.8	2.5[e10]	French et al., 1995	Meso-tidal	Lagoon[c]	15/7/2017
Krabbenkreek, Netherlands	S4	salt marsh	51°35' N, 4°8'E	31N	3.3	-	1.7[e11]	van Eerdt, 1985	Meso-tidal	Estuary[a]	8/7/2018
Verdronken Land van Saeftinghe, Netherlands	S5	salt marsh	51°20' N, 4 9'E	31N	4.8	-	2.2[e12]	Kornman, 1991	Macro-tidal	Estuary[a]	6/8/2018
Crystal River, USA	S6	salt marsh	28°54' N, 82 36'W	17N	0.6	-	0.6[e13]	Frazer, 2006	Micro-tidal	Open/ Bay[b]	8/8/2018
Chongming Island, China	S7	salt marsh	31°29'0.32''N, 121°57'39.55''E	51N	2.3		0.9[e14]	Shi et al. 2017	Meso-tidal	Estuary[e14]	01/10/2021
Great Marsh, DE, US	S8	salt marsh	38°47'11.95''N, 75°11'24.62''W	18N	1.0		0.7[e15]	Stumpf 1983	Micro-tidal	Estuary[e14]	24/09/2021

Supplementary Table 1. Overview of investigated systems around the world: Site characteristics of the selected locations for spectral image analysis, mtl – mean tidal range, mstl – mean spring tidal range, elev.(m rel. msl) – elevation in meter relative to mean sea level; *https://maps.coastalresilience.org/mangrove-restoration/#; [a]https://data.unep-wcmc.org/datasets/23; [b]Jackson, K., & Pluckhahn, T. J. (2020). A First Millennium AD Vegetation History from the Crystal River Site (8CI1), Florida. The Journal of Island and Coastal Archaeology, 15(1), 57-79; [c]French, Jonathan R., and Tom Spencer. "Dynamics of sedimentation in

a tide-dominated backbarrier salt marsh, Norfolk, UK." Marine Geology 110.3-4 (1993): 315-331.; [d]Silvestri, S., Defina, A., & Marani, M. (2005). Tidal regime, salinity and salt marsh plant zonation. Estuarine, coastal and shelf science, 62(1-2), 119-130. [e]Lefeuvre, Jean-Claude, et al. "European salt marshes diversity and functioning: the case study of the Mont Saint-Michel bay, France." Wetlands Ecology and Management 8.2 (2000): 147-161; [e1]Dittel et al., 1991, https://doi.org/10.1016/0272-7714(91)90010-9; [e2]Minderhoud, et al., 2019, https://doi.org/10.1038/s41467-019-11602-1; [e3]Metcalfe et al., 2011 (https://doi.org/10.1016/j.ecss.2011.09.006); [e4]Pelckmans, Ignace, et al. "Hydrodynamic modelling of the tide propagation in a tropical delta: overcoming the challenges of data scarcity." Proceedings of the papers submitted to the 2020 TELEMAC-MASCARET User Conference October 2021; [e5]Perera et al., 2019, https://doi.org/10.1016/j.geoderma.2019.03.041; [e6]Murray, 1994 (report: https://pubs.usgs.gov/of/1994/0116/report.pdf; [e7]Horstman, Erik M., Karin R. Bryan, and Julia C. Mullarney. "Drag variations, tidal asymmetry and tidal range changes in a mangrove creek system." Earth Surface Processes and Landforms (2021). [e8]Weill et al., 2012(https://doi.org/10.1016/j.sedgeo.2010.12.002); [e9]Carniello et al., 2009 (https://doi.org/10.1029/2008JF001157); [e10]Stoddart et al., 1989 (https://doi.org/10.2307/1351902); [e11]Ma et al., 2014 (https://doi.org/10.1016/j.ecss.2014.05.001), [e12]Stark et al., 2016 (https://doi.org/10.1016/j.ecss.2016.03.027); [e13]Marth, Nancy J., et al. Centimeter-Level Orthometric Heights at Reference Points Along Florida's Big Bend Coastline from Global Positioning System (GPS) Static Surveys. No. 95-216. US Geological Survey: Center for Coastal Geology [distributor], 1995. [e14]Zhu, Zhenchang, et al. "Interactions between the range expansion of saltmarsh vegetation and hydrodynamic regimes in the Yangtze Estuary, China." Estuarine, Coastal and Shelf Science 96 (2012): 273-279; [e15]Stumpf, Richard P. "The process of sedimentation on the surface of a salt marsh." Estuarine, Coastal and Shelf Science 17.5 (1983): 495-508; Shi, B. W., et al. "Role of wind in erosion- accretion cycles on an estuarine mudflat." Journal of Geophysical Research: Oceans 122.1 (2017): 193-206. Stumpf, Richard P. "The process of sedimentation on the surface of a salt marsh." Estuarine, Coastal and Shelf Science 17.5 (1983): 495-508.

ID	Dite	Lat.	Lon.	Main species	Source
**M1**	Gulf of Nicoya, Costa Rica	10^{0}7'N	85^{0}15'W	Rhizophora racemosa and Avicennia germinans	Li et al Poster
**M2**	Mekong, Vietnam	9^{0}49'N	106^{0}38'E	Sonneratia and Avicennia	Bullock et al., 2017
**M3**	Darwin Harbour, Australia	12^{0}26'S	130^{0}52'E	Sonneratia alba, Rhizophora stylosa and Ceriops tagal	Rogers et al., 2017
**M4**	Churute, Ecuador	2^{0}37'S	79^{0}45'W	Rhizophora mangle, Avicennia germinans, Laguneularia racemosa and Conocarpus erectus	Gara et al., 1990
**M5**	Kala Oya, Sri Lanka	8^{0} 17' N	79^{0} 50' E	Avicennia marina, Rhizophora mucronata, Ceriops tagal and Lumnitzera racemosa	Perera et al., 2013
**M6**	Goodland, USA	25^{0} 56' N	81^{0} 35' W	Rhizophora mangle, Avicennia germinans and Laguncularia racemosa	Sengupta et al., 2005
**M7**	Whitianga, New Zealand	36°52'56" S	175°42"2' E	Avicennia marina var. australasica	Horstman et al., 2021

Supplementary Table 2. Overview of investigated mangrove systems and occurring species

Source:

Venegas-Li, Rubén & Morales, Lucia & Martínez-Fernández, Damián. (2013). Mapping Mangrove Species Composition with Rapideye Satellite Images in the Nicoya Gulf, Costa Rica: How far can we go? *ASSOCIATION FOR TROPICAL BIOLOGY AND CONSERVATION (ATBC).*

Bullock, E. L., Fagherazzi, S., Nardin, W., Vo-Luong, P., Nguyen, P., and Woodcock, C. E. (2017), Temporal patterns in species zonation in a mangrove forest in the Mekong Delta, Vietnam, using a time series of Landsat imagery, *Continental Shelf Research*, 147, 144-154.

Rogers, K., Lymburner, L., Salum, R., Brooke, B. P., and Woodroffe, C. D. (2017), Mapping of mangrove extent and zonation using high and low tide composites of Landsat data, *Hydrobiologia*, 803(1), 49-68.

Gara, R. I., Sarango, A., & Cannon, P. G. (1990). DEFOLIATION OF AN ECUADORIAN MANGROVE FOREST BY THE BAGWORM, OIKETICUS KIRBYI GUILDING (LEPIDOPTERA: PSYCHIDAE). *Journal of Tropical Forest Science*, 3(2), 181–186.

Perera, K. A. R. S., Amarasinghe, M. D., and Somaratna, S. (2013), Vegetation structure and species distribution of mangroves along a soil salinity gradient in a micro tidal estuary on the North-western Coast of Sri Lanka, *American Journal of Marine Science,* 1(1), 7-15.

Sengupta, R., Middleton, B., Yan, C., Zuro, M., and Hartman, H. (2005), Landscape characteristics of Rhizophora mangle forests and propagule deposition in coastal environments of Florida (USA), *Landscape Ecology*, 20(1), 63-72.

Horstman, E. M., Bryan, K. R., and Mullarney, J. C. (2021), Drag variations, tidal asymmetry and tidal range changes in a mangrove creek system, *Earth Surface Processes and Landforms*, 46(9), 1828-1846.

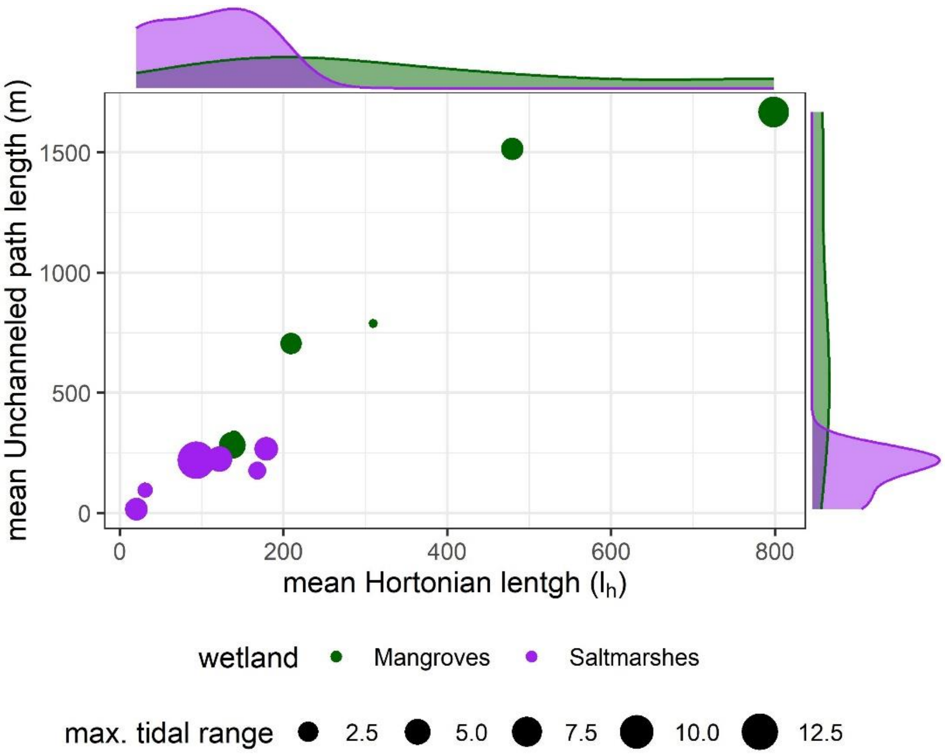

Supplementary Figure 3. Mean unchanneled path length, mean Hortonian length (*lh*) and tidal range: The constant linear slope between mean unchanneled path length and mean Hortonian length, indicates a similar geometric efficiency, which is not dependent on tidal range.

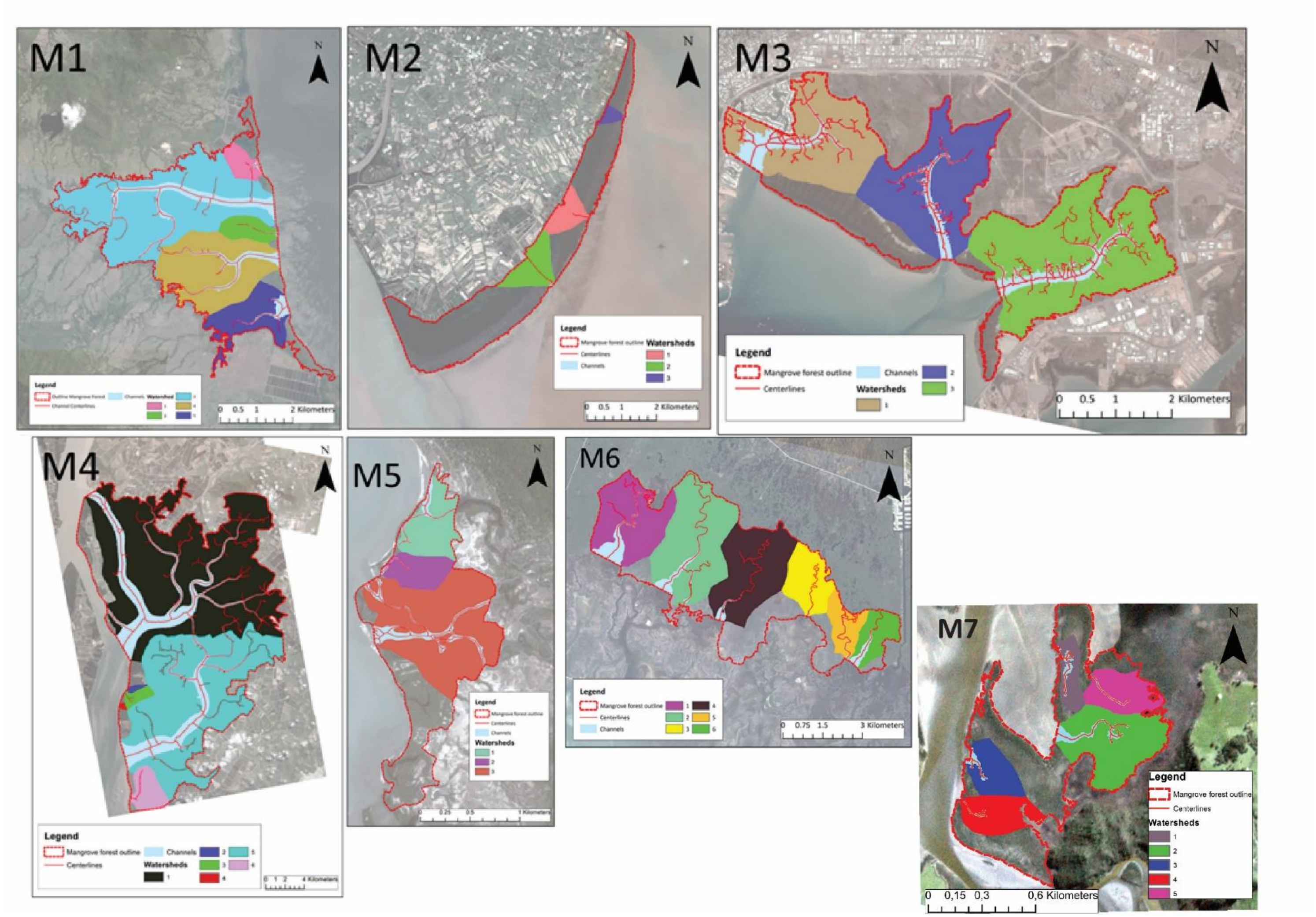

Supplementary Figure 4. Overview Mangrove Systems: ID's M1-M7 represent the systems analysed for channel networks, details see table S1. Channels are depicted by red continuous lines; the mangrove forest outline is displayed by red dashed lines; watersheds are coloured polygons; the satellite image used for channel extraction is shown in the background.

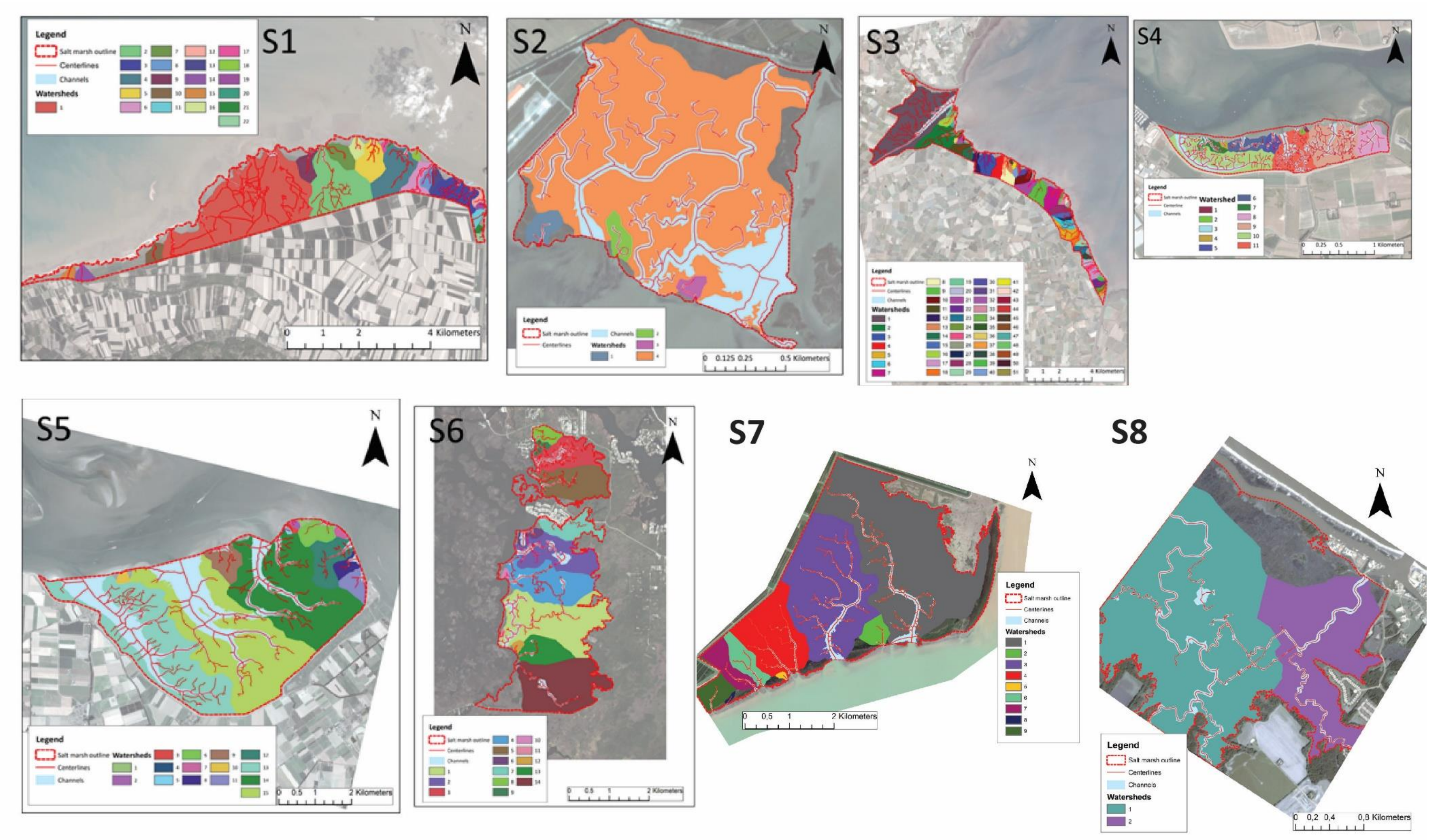

Supplementary Figure 5 Overview Salt marsh Systems: ID's S1-S8 represent the systems analysed, for details see table S1. Channels are depicted by red continuous lines; the salt marsh outline is displayed by red dashed lines; watersheds are coloured polygons; the satellite image used for channel extraction is shown in the background.

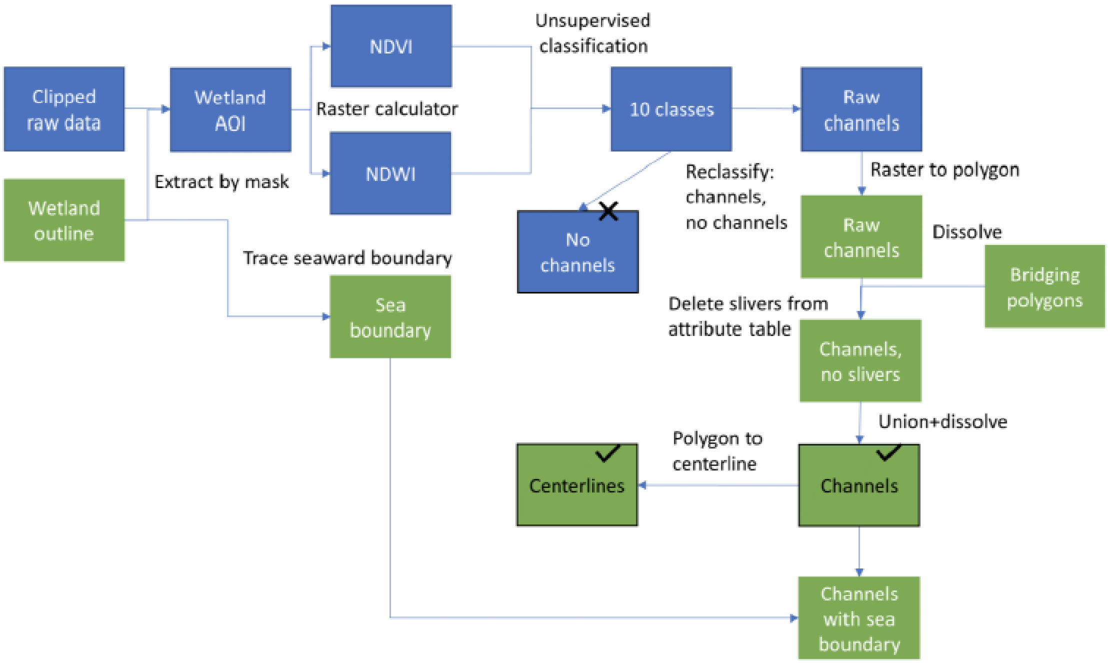

Supplementary Figure 6. Channel extraction: First, the mangrove/salt marsh outline was extracted from the clipped raw data. Two indices (NDVI and NDWI) were used as input for an unsupervised classification, using the maximum likelihood classifier. Pre-tests determined the optimal number of classes to be ten. The resulting ten classes were manually reclassified into two distinct classes: 1) channels and 2) no channels. Whether the class was reclassified as channel was based on context (flowing into the larger water body) and shape (elongated and bifurcating). The resulting raster was conversed to vector polygon in order to dissolve bridging polygons into the raw channels to generate an interconnected network. Since all individual channel parts were connected, small wet parts (<600m^2) disconnected from the network were deleted. The resulting vector polygon contained the uninterrupted outline of channels but still contained gaps within the network, which was solved by a union overlay and a subsequent dissolve, prohibiting gaps. The resulting 'channels' vector polygon was conversed to polylines that were situated in the center of the polygon. This was done by 'polygon to centerline' by the XtoolsPro extension for ArcGIS desktop. In some cases, these centerlines needed manual improvement when channels met the boundaries of the marsh/mangrove forest, see Figure S7.

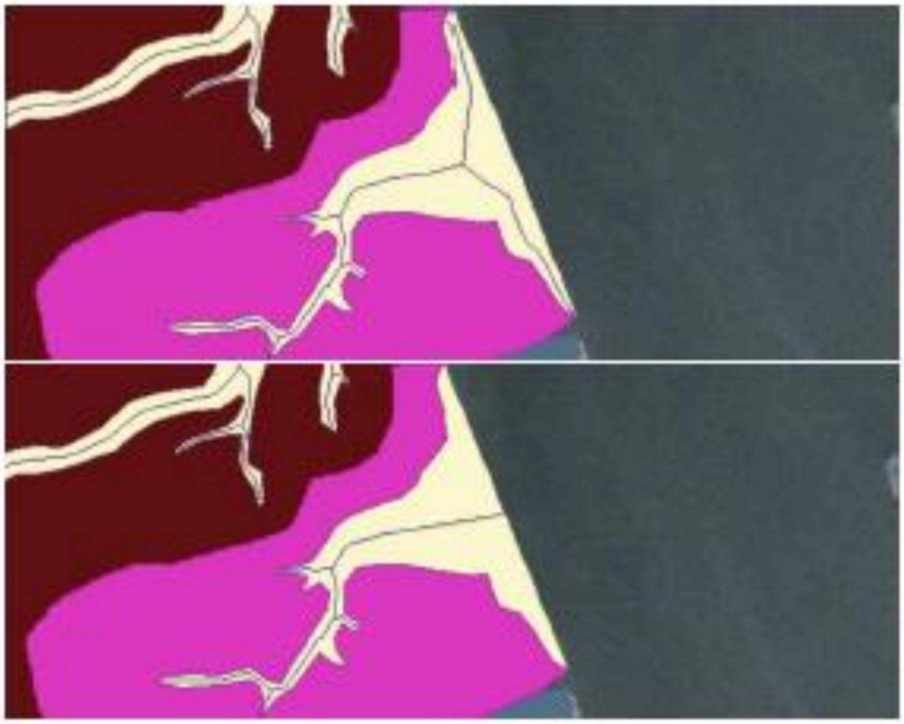

Supplementary Figure 7. Channel optimization: Manual improvement of the centerlines exemplified at the centerline network of het Verdronken Land van Saeftinghe, The Netherlands. The upper panel shows the unimproved centerlines; the lower panel depicts the improved centerlines.

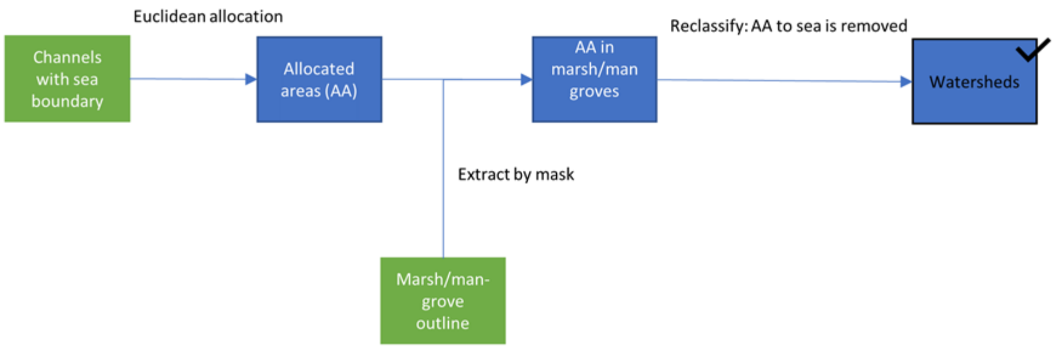

Supplementary Figure 8. Watershed delineation: First, a raster is composed using the euclidean allocation algorithm, allocating pixels to channels and the seaward boundary of the system. The mangrove/salt marsh area is extracted from these allocated areas (AA). A reclassification is used to delete the pixels that are allocated to the seaward boundary.

Method validation and sensitivity:

Mangrove and salt marsh channelization derived from remote-sensing data might produce inaccurate estimates of channel metrics due to the growth physiology of the wetland vegetation and limits in satellite data resolution. More specifically, above-ground biomass of salt marsh grasses or the crowns of mangrove trees might prevent the detection of narrow channels. For this reason, we carried out a sensitivity study and data correction procedures: (1) We compared the extracted channel metrics mean unchanneled path length (mUpl) and drainage density (DD) with existing ground truthing data for 1 mangrove (Whitianga, New Zealand) and 1 salt marsh case (Saeftinghe, the Netherlands). Mangrove ground truthing data was provided by Horstman et al. (2021), salt marsh ground truth data was available at the Dutch authorities (Rijkswaterstaat, RWS.nl). (2) We subsequently removed 1st – 5th order of Hortonian channels from the extracted DEM networks and recalculated the channel metrics to investigate the impact of undetected channels on the extracted metrics (Fig. 9a-c). This analysis revealed that our satellite-based method consistently fails to capture 1st order channels which have an average-width of 8.21 m and 7.9 m for Saeftinghe and Whitianga DEM networks, respectively. In other words, channel metrics extracted from satellite images matched channel metrics of the DEM without 1st order channels (Fig.9c). (3) Subsequently, we established power law relationships between channel order and respective mean channel metrics and calculated correction factors for mUpl and DD for salt marshes and mangroves accounting for missing 1st order channels (Fig. 9b); $Corr_{mangroveDD}$: 0.548; $Corr_{saltmarshDD}$: 0.463; $Corr_{saltmarshMUPL}$: 2.03; $Corr_{mangroveMUPL}$: 1.61. The correction factors were subsequently applied to the extracted channel metrics. To also correct for the channel lengths presented in Fig.3, we employed the same methodology for the cumulative channel length ($Corr_{mangroveLENGTH}$: 0.481; $Corr_{saltmarshLENGTH}$: 0.467), to investigate the scaling of DD. This assumes the spatial-scaling stays constant across scales as shown by Marani et al. (2003).

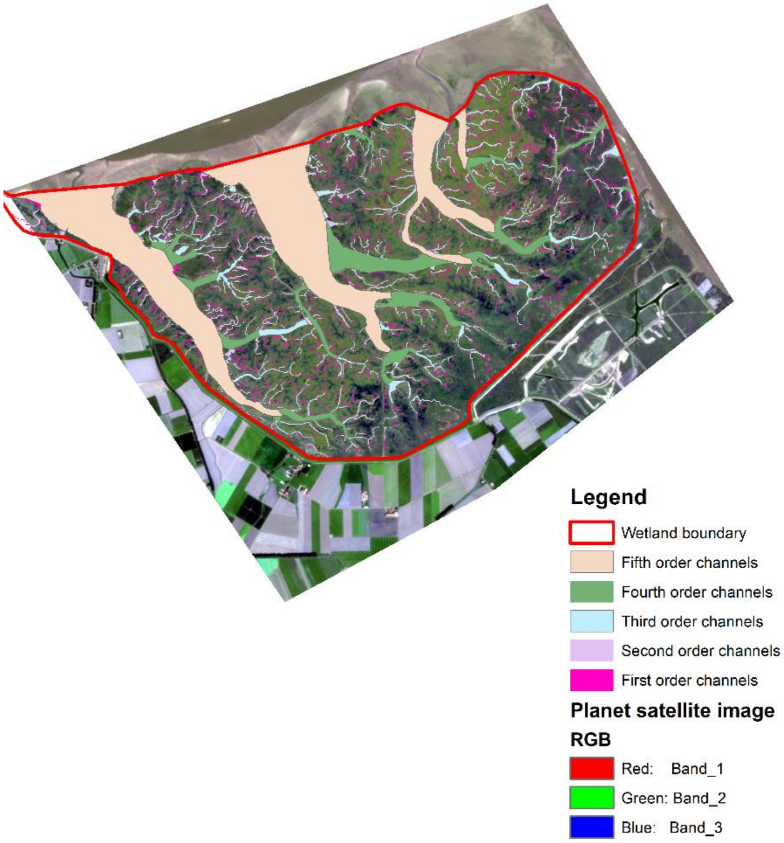

Supplementary Figure 9a. Sensitivity test on channel metrics for salt marshes with the example of the Saeftinghe salt marsh, the Netherlands. Channels are extracted based on an existing digital elevation model of Rijkswaterstaat, different colors of the channels show the different Hortonian channel orders removed for the sensitivity test.

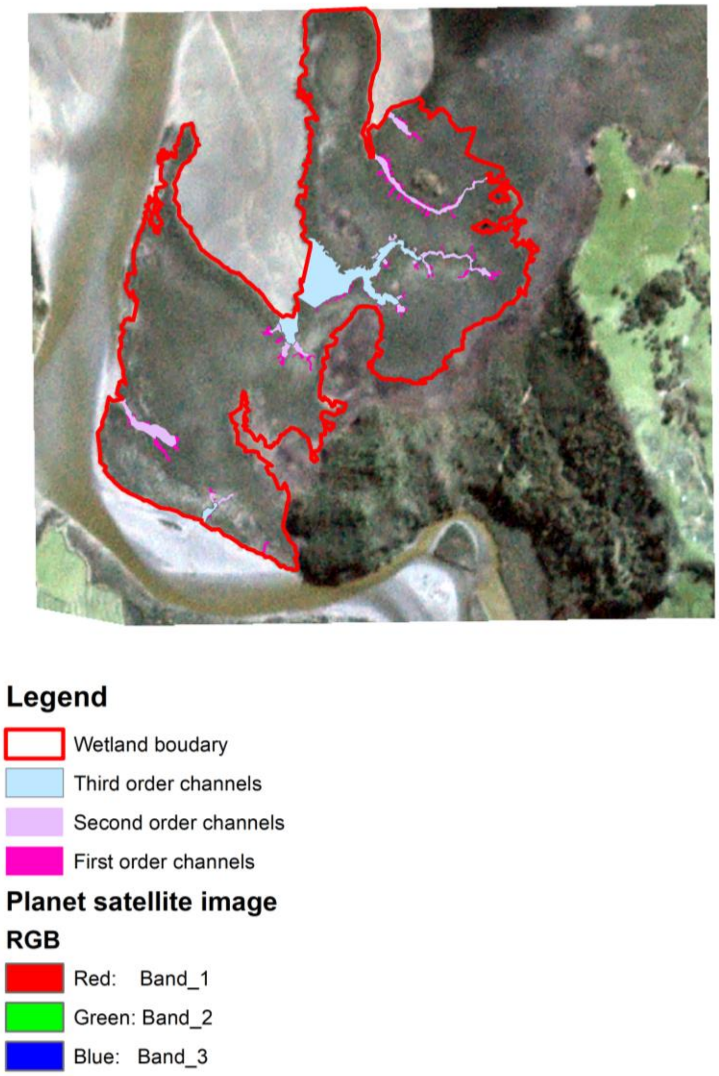

Supplementary Figure 9b. Sensitivity test on channel metrics for mangroves with the example of the Whitianga mangrove forest, New Zealand. Channels are extracted based on an existing digital elevation model. Different colors of the channels show the different Hortonian channel orders removed for the sensitivity test.

Horstman, Erik M., Karin R. Bryan, and Julia C. Mullarney. "Drag variations, tidal asymmetry and tidal range changes in a mangrove creek system." Earth Surface Processes and Landforms (2021)

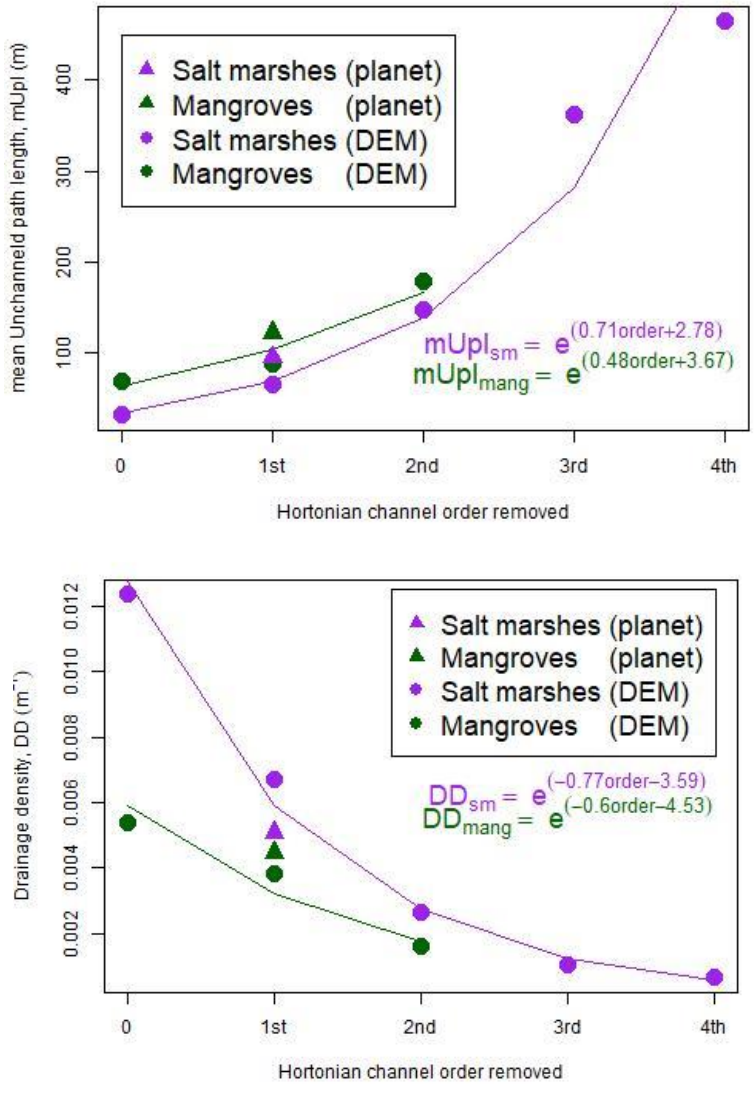

Supplementary Figure 9c. Sensitivity and correction. Impact of removing channel orders on mean unchanneled path length and drainage density. Data extracted from Planet Labs satellite imagery correspond with the DEM data set when the first order channels are removed (comparison between dots and triangles). The power law relationship for the removed channel orders was then used to correct the Planet Labs data. We used the correction factor between orders 1 and 0 predicted by the model on the respective equation per wetland type and variable. Triangles represent the satellite based channel network; circles represent the digital elevation based channel network; green represents mangroves; purple represents salt marshes

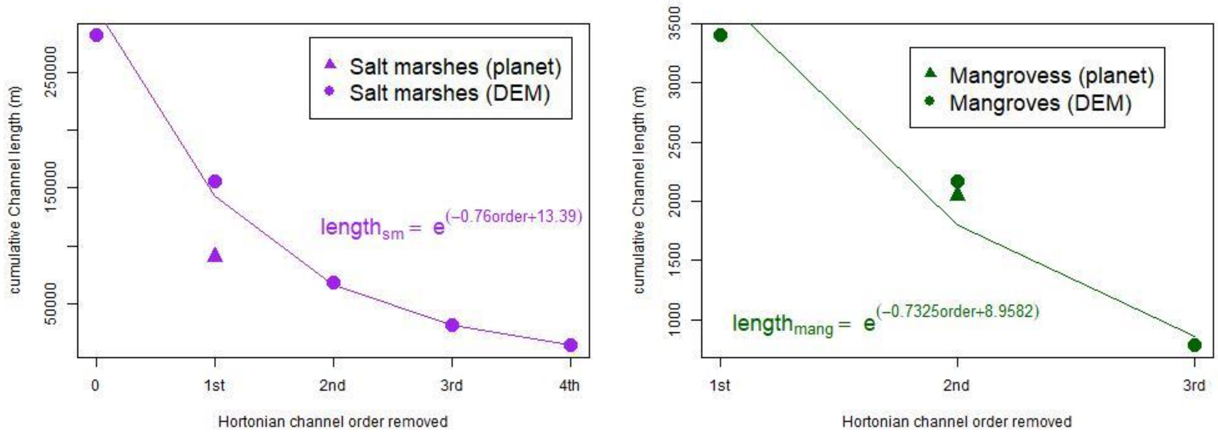

Supplementary Figure 9d. Sensitivity and correction. Impact of removing channel orders on mean unchanneled path length and drainage density. Data extracted from Planet Labs satellite imagery correspond with the DEM data set when the first order channels are removed (comparison between dots and triangles). The power law relationship for the removed channel orders was then used to correct the Planet Labs data. We used the correction factor between orders 1 and 0 predicted by the model on the respective equation per wetland type and variable. Triangles represent metrics from the satellite based channel network; circles represent metrics from the digital elevation based channel network; green represents mangroves; purple represents salt marshes; we applied the correction on the cumulative channel length and plotted the corrected data in fig.3.

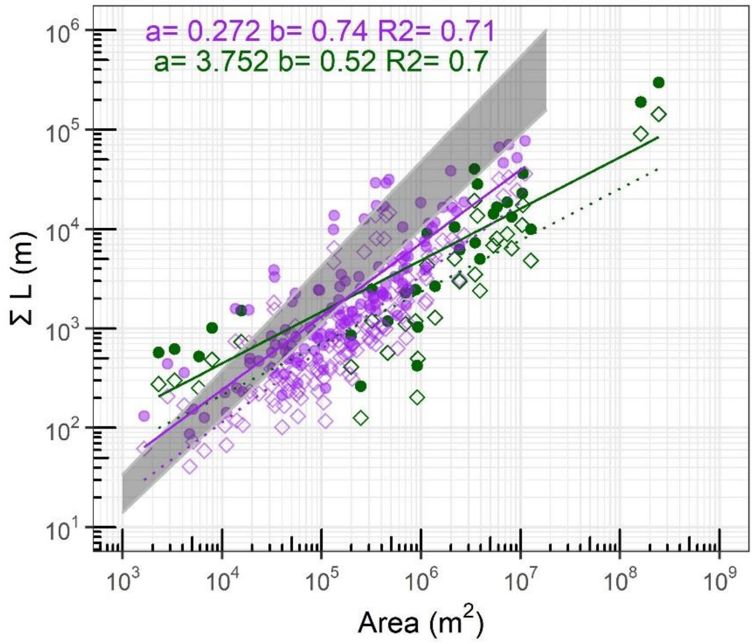

Supplementary Figure 9e. Correction Channel Length. Shows a comparison between the uncorrected (◊, diamond) and corrected (●, dots) channel lengths over drainage area.The comparison reveals that especially for the salt marshes (purple color) the re-scaled values approach scaling relationships derived from Digital Terrain Models in the Venice lagoon (grey shaded area) which supports to applicability of the calculated correction factors.

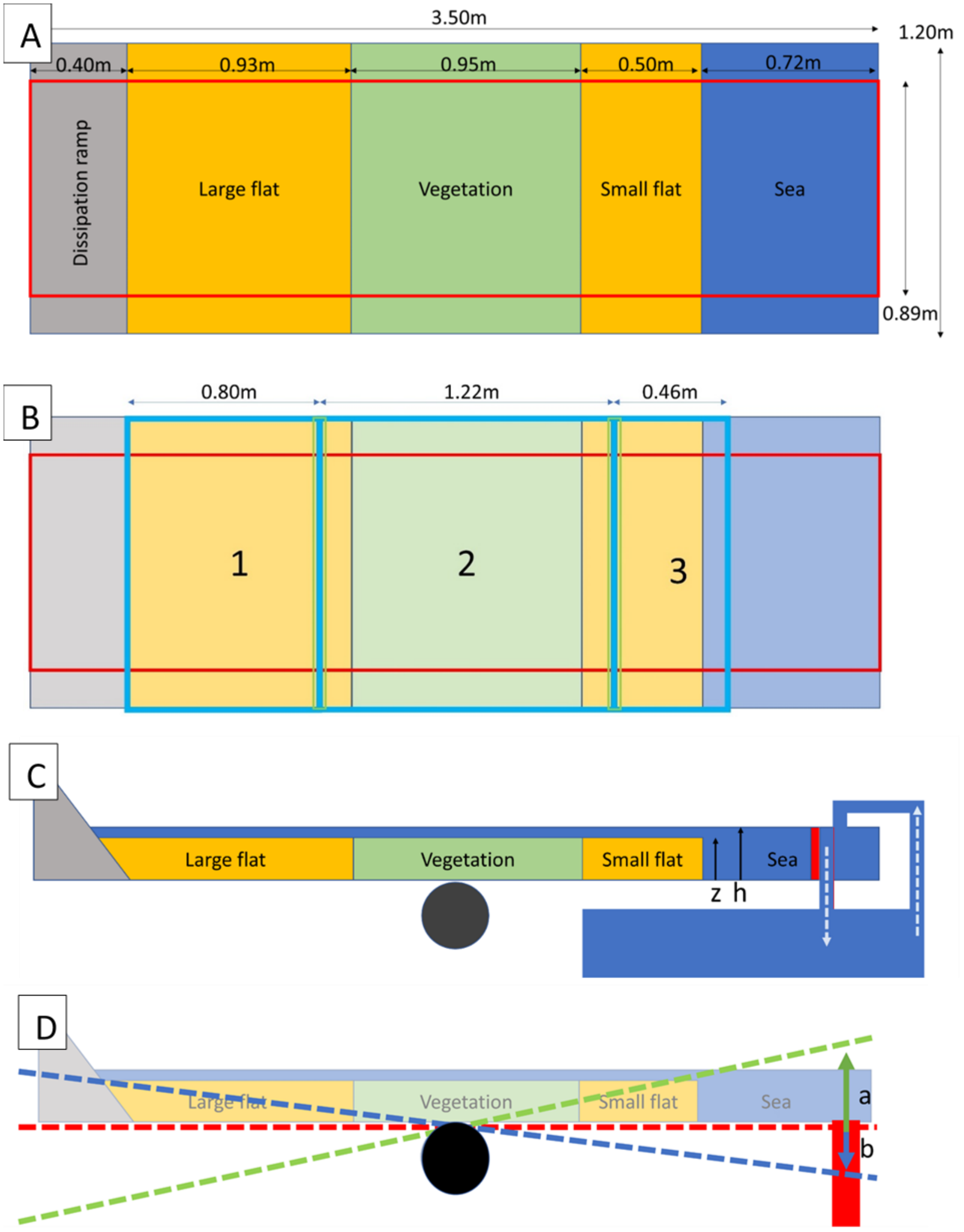

Supplementary Figure 10. Experimental setup: Schematic overview of used tilting flume. Panel A depicts the dimensions of distinct sections of the set-up: the artificial sea is located on the right depicted in blue; the vegetation/colonization zone, is depicted in green in the middle of the flume bordered by a small sandy flat on the left and a large flat on the right side. The large flat was set to have enough space to accelerate the ebb flow through the vegetation. The small flat serves as a tidal flat that connects the sea with the artificial colonization zone as in nature. This set-up is comparable to a colonized chenier or sand bar in nature. Panel B depicts the sublayer of

detachable malls: 1, 2 and 3. Panel C shows a water reservoir, the flow generated by the pump (light blue dotted arrows), water level (h), bed level (z) and an overflow pipe determine the water level (red rectangle). Panel D shows the tilted plane during high water with respect to the horizontal plane (dotted red line) as a dotted green line with tilt amplitude a. The tilted plane during low water with respect to the horizontal plane is depicted as the blue dotted line with tilt amplitude b. The mechanical jack facilitating the tilt is depicted as a red bar in the lower panel; the tilt axis is depicted as the black circle in the lower panel.

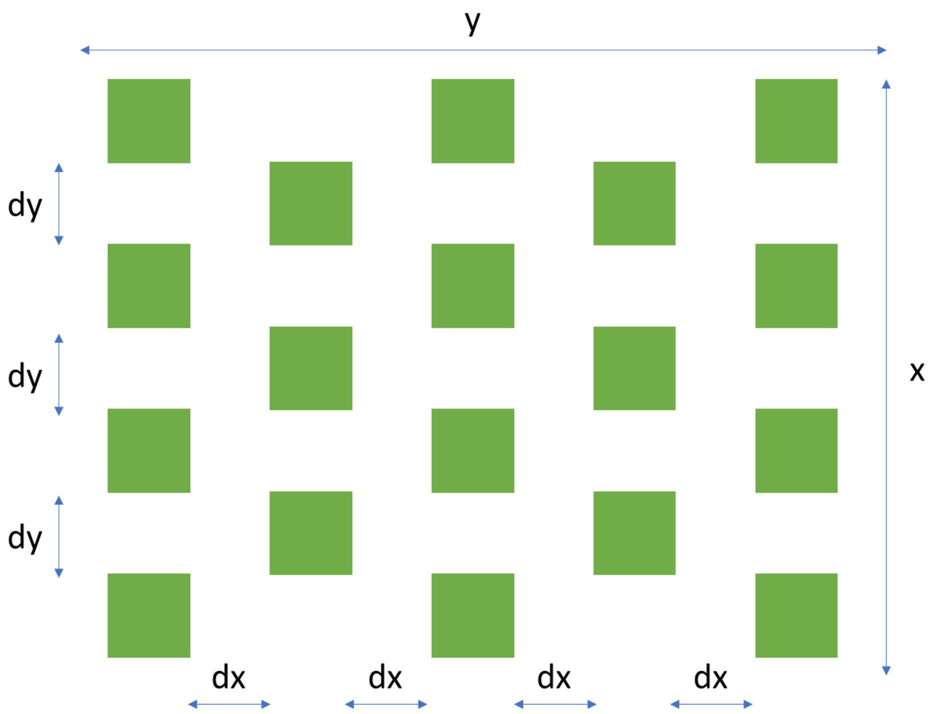

Supplementary Figure 11. Interpatch distances: Scheme of interpatch distances dx and dy, the y- and x direction, see Supplementary Table 3.

Configuratio n	Vegetation	Patch diameter (m)	dy (m)	dx (m)	Max. amplitude a (m)	Max amplitu de b (m)	Water level (m)	Period(s)	Total amount of cycles
homogenous	Med. sat.	-	-	-	0.01	0.07	0.045	112.5	2880
patches	Med. sat	~ 0.10	~ 0.10	~ 0.10	0.01	0.07	0.045	112.5	1330

Supplementary Table 3. Experimental parameters: For both configurations *Medicago sativa* was used. Patch diameter, interpatch distance in y and x (dy, dx, see Figure S5) were only displayed for the patchy configuration and are approximate, since patch edges are not absolute due to seed germination and dispersion. No patches were used in the homogenous configuration. Amplitudes a and b reflect amplitude in Figure S4. For both configurations the same abiotic conditions were used (water level and period). Total amount of cycles is less for the patchy configuration, because morphological equilibrium established faster compared to the homogenous configuration.
